# Central Nervous System Involvement of Epstein Barr Virus Associated Lymphoproliferative Disorder in a Child with Acute Lymphoblastic Leukemia: Successful Treatment with Rituximab and Interferon-Alpha

**DOI:** 10.4274/tjh.2011.0047

**Published:** 2013-03-05

**Authors:** Berna Atabay, Meral Türker, Can Öztürk, Sümer Sütçüoğlu, Haldun Öniz, Esra Arun Özer

**Affiliations:** 1 Tepecik Training and Research Hospital, Department of Pediatrics Clinic, İzmir, Turkey

**Keywords:** Epstein-Barr virus, Lymphoproliferative disorder, Rituximab, children, Leukemia

## Abstract

Central nervous system (CNS) involvement of Epstein-Barr virus (EBV)-associated lymphoproliferative disease is a rare and serious complication in children with leukemia. Although rituximab therapy seems to be promising in these cases, persistent hypogammaglobulinemia may appear after treatment due to complete depletion of normal B lymphocytes in the peripheral blood. Here we report isolated CNS involvement of EBV-associated lymphoproliferative disorder in a 4-year-old boy with acute leukemia. The patient was treated with rituximab and interferon alpha; however, persistent hypogammaglobulinemia developed as a complication. Given the rarity of the complication in children receiving these agents, our experience with such a case may be helpful to others.

**Conflict of interest:**None declared.

## Abstract (tr)

Santral sinir sisteminin EBV ilişkili lenfoproliferatif hastalığı nadir bir durum olup, çocukluk çağı lösemilerinin ciddi bir komplikasyonudur. Bu olgularda rituximab tedavisi faydalı olmakla birlikte, periferik kanda normal B lenfositlerinin tam kaybı sonucunda persistan hipogamaglobulinemi ortaya çıkabilmektedir. Burada akut lösemili 4 yaşında bir erkek çocukta, EBV ilişkili lenfoproliferatif hastalığın izole santral sinir sistemi tutulumu sunulmaktadır. Hasta rituximab ve alfa interferon ile tedavi edilmiş, ancak komplikasyon olarak persistan hipogamaglobulinemi gelişmiştir. Bu ilaçları kullanan çocuklarda nadir görülen bir komplikasyon olması nedeniyle, bu olgu ile elde ettiğimiz deneyimin diğerlerine yardımcı olacağını düşünmekteyiz.

## INTRODUCTION

Epstein-Barr virus (EBV)-associated lymphoproliferative disorder (LPD) is a severe and fatal complication of patients with immune deficiency [1]. The clinical picture of EBV-associated LPD is quite variable, and isolated central nervous system (CNS) involvement is rare. There are scanty data in the literature concerning the management of this disorder. Rituximab is a monoclonal antibody against CD20-positive B cells and is widely used in EBV-related lymphomas and diseases [2].

Here we report isolated CNS involvement of EBV-associated LPD in a 4-year-old boy with acute leukemia. The patient was treated with rituximab; however, hypogammaglobulinemia developed as a complication. Given the rarity of the complication in children receiving this agent, our experience with such a case may be helpful to others.

## CASE REPORT

A 4-year-old boy was referred to our hospital in February 2007 for the treatment of biphenotypic acute leukemia. There was no family history of immunodeficiency, fatal infectious mononucleosis, or lymphomas. At the time of diagnosis he had no blasts in the cerebrospinal fluid (CSF) examination. The bone marrow aspirate confirmed the diagnosis of biphenotypic acute leukemia. His initial diagnostic flow cytometry results were: CD45, 72.5%; CD34, 71.8%; CD19, 77.9%; CD10, 72.5%; CD22, 69.4%; CD7, 15.8%; CD33, 72.7%; CD13, 76.3%; CD3, 13.3%; CD2, 18.8%; CD15, 4.7%; CD117, 4.1%; MPO, 10.3%; TdT, 42.7%; CD10+CD19, 71.6%. Cytogenetic analysis of his bone marrow was normal. His serum immunoglobulin levels were: IgA, 83 mg/dL (normal value: 14-159 mg/dL); IgM, 161 mg/dL (normal value: 43-207 mg/dL); and IgG, 1150 mg/dL (normal value: 345-1236 mg/dL). Lymphocyte subsets were not calculated initially. EBV serology was positive for viral capsid antigen (VCA) IgG, negative for VCA IgM, and consistent with a prior EBV infection. The patient was enrolled in a Berlin-Frankfurt-Munster–derived protocol [3]. Complete remission was attained after induction and was maintained with consolidation. 

Maintenance therapy was initiated in October 2007, consisting of daily 6-mercaptopurine and weekly methotrexate administered orally on an outpatient basis. No cranial irradiation was administered. On the second month of maintenance therapy, the patient presented with a 3-day history of fever, malaise, and sore throat. Physical examination was unremarkable except for high fever (39 ^°^C) and whitish tonsillar exudate. Maintenance therapy was ceased and systemic antibiotic therapy was administered. Three days after therapy, the patient was better and afebrile. 

**Figure 1:**
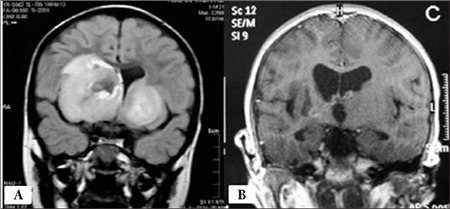
A) MRI of the brain showing 2 huge masses in the periventricular area. Mild brain edema was seen around the lesion. B) MRI showed complete resolution of the masses after treatment.

After 3 weeks, the patient presented with vomiting, diarrhea, and high fever (39 ^°^C). Physical examination was normal. Laboratory tests showed hemoglobin of 9.4 g/dL, white blood cell count of 2 × 109/L with 43% segmented neutrophils, 3% bands (absolute neutrophil count of 860/µL), 4% eosinophils, 16% monocytes, and 34% lymphocytes. Blood biochemical profile and chest X-ray were normal. The child was admitted to the hospital for antibiotic therapy. On the 10^th^ day of hospitalization, he presented with left hemiparesis and central facial paresis. Brain magnetic resonance imaging (MRI) revealed 2 masses located in the periventricular area (Figure 1A). Bone marrow aspirate and CSF examination were not suggestive of a leukemic relapse. Thorax and abdomen computerized tomography (CT) were unremarkable. Serum EBV VCA IgM and IgG were positive, whereas anti-Epstein-Barr nuclear antigen IgM and p22 antigen were negative. Human immunodeficiency virus and cytomegalovirus serology were also negative. Serum immunoglobulin levels were normal. The stereotactic brain biopsy showed an atypical lymphoid infiltrate composed predominantly of CD79a-positive B cells involving brain parenchyma with a prominent perivascular distribution. The infiltrate was negative for CD10, CD34, TdT, MPO, CD117, and lysozyme. The biopsy was positive for EBV-encoded small nonpolyadenylated RNAs by in situ hybridization test. The patient was diagnosed with localized EBV-associated LPD and administered weekly anti-CD20 monoclonal antibody (rituximab, 375 mg/m^2^) for 4 weeks and alpha-interferon 3 times per week (3 × 106 units/m^2^) for 6 months due to the infiltration of CD20-positive B cells. The therapy was well tolerated and the patient’s neurologic condition did not show deterioration. After therapy serum immunoglobulin levels were low (IgG, 200 mg/dL; IgM, 8 mg/dL; and IgA, <6 mg/dL). The lymphocyte subset panel was normal (CD19, 12%; CD20, 18%; CD3, 76%; CD4, 28%; CD8, 4%; and CD16+56, 15%). The patient was administered monthly intravenous immunoglobulin at 0.5 g/kg to achieve at least 500 mg/dL IgG levels. After 2 months of therapy, the patient’s neurological signs had disappeared. Cranial MRI showed complete resolution of the masses (Figure 1B).

For the subsequent 3 years of follow-up, the patient received no chemotherapy. The disease is in remission but intravenous immunoglobulin therapy is still given every 4 or 6 weeks because of IgG deficiency. 

## DISCUSSION

EBV-associated LPD is an uncommon and often fatal complication of patients with inherited or acquired immune deficiency [4]. Its clinical presentation is marked by serologic evidence of active EBV proliferation, attributable either to a primary EBV infection or the reactivation of a latent infection. There are 10 cases of EBV-associated LPD in children with acute leukemia previously reported [5,6,7,8,9,10,11,12]. Of those, 2 cases were presented with CNS involvement. The age at diagnosis of the cases ranged between 3 to 16, and 3 patients died. Most of the patients treated by Bernard et al. received supportive therapy, whereas only one patient was given rituximab [5]. Despite good response to the therapy, that patient died with relapsed leukemia after 6 months. Incidence of brain involvement is more common in cases with EBV besides acute lymphoblastic leukemia [13].

Different treatments for EBV-associated LPD have been proposed. Therapies have targets including the decrease of immunosuppression and improvement of immune function, or the removal of the lymphoproliferative lesion. Another strategy is to eliminate EBV-infected B cells with chemotherapy or antiviral drugs or with the infusion of polyclonal donor-derived T cells specific for EBV proteins in the bone marrow of transplant recipients [14]. Recently the use of monoclonal anti-B cell antibodies has been proposed because of the expression of these antigens on the surface of EBV-transformed B cells [5,15]. 

Our patient received rituximab because the infiltration consisted predominantly of CD20-positive B cells. His neurological signs disappeared and cranial MRI showed complete resolution of the masses. Since rituximab targets normal B cells as well as neoplastic B cells, almost complete depletion of normal B lymphocytes in the peripheral blood is observed for an average of 6 to 9 months after starting the therapy [16,17,18]. However, hypogammaglobulinemia related to B cell depletion after rituximab treatment is not usually problematic and is not associated with any clinical morbidity. Recently a prolonged hypogammaglobulinemia was reported in some patients receiving the therapy as an adjuvant to autologous stem cell transplantation (Table 1) [19,20]. Additionally in most cases, the level of hypogammaglobulinemia is mild, although it sometimes persists for over 2 years. Irie et al. [21] reported a 60-year-old woman diagnosed with follicular lymphoma who received rituximab therapy. She developed hypogammaglobulinemia persisting for 6 years. Nishio et al. [22] analyzed the phenotypes of B cells in patients receiving rituximab and showed that recovery of memory B cells was delayed and naive B cells failed to differentiate into memory cells or plasma cells upon stimulation with SAC, IL-2, IL-10, and CD40L in vitro. In addition, they reported that FC-gamma-RIII-alpha gene polymorphism was related to the immunoglobulin level. We think that the naive B cells of our patient also failed to differentiate into memory B cells or plasma cells. 

Although the reason for B cell differentiation arrest is still unclear, there are 2 possible explanations for this phenomenon. First, genetic aberration of a factor essential for immunoglobulin rearrangement may have occurred in lymphoid progenitor cells after combination chemotherapy. Second, rituximab combined chemotherapy may be associated with a risk of persistent differentiation arrest and apoptosis of B cell lineage in patients with a specific genetic background [21]. Since our patient was also treated with alpha-interferon and chemotherapy, those might have contributed to hypogammaglobulinemia. Because interferon is widely used for decreasing immunosuppression and improving immune function, it is also speculated that alpha-interferon may have contributed to remission of leukemia in this case [23].

X-linked lymphoproliferative disease is a rare immunodeficiency disease that is characterized by a prediction for fatal or near-fatal EBV-induced infectious mononucleosis in childhood, subsequent hypogammaglobulinemia, and a markedly increased risk of lymphoma or other lymphoproliferative diseases [24]. A definitive diagnosis of X-linked lymphoproliferative disease is with mutation analysis for the SH2D1A and XIAP genes mutation. However we did not exclude the diagnosis of X-linked lymphoproliferative disease because the mutation analysis could not be performed in our case. 

In conclusion, EBV-associated LPD in leukemia is a serious complication and extremely rare clinical entity. The present case was treated successfully with rituximab and alpha-interferon. Although rituximab therapy seems to be promising in these cases, persistent hypogammaglobulinemia may appear after treatment. Physicians treating this disease with rituximab should be aware of this rare potential complication. The chemotherapy and alpha-interferon treatment may contribute to hypogammaglobulinemia. In addition, further studies are needed to elucidate the pathogenesis of hypogammaglobulinemia after rituximab administration.

## Figures and Tables

**Table 1:**
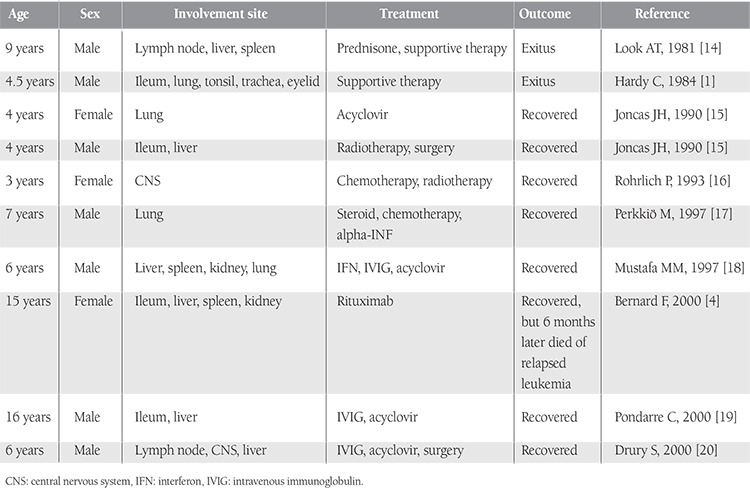
Clinical data of the published cases with acute leukemia that had development of EBV-associated lymphoproliferative disorder.
